# Supplementary material for: A Critical View of the Application of the APEX Software (Aqueous Photochemistry of Environmentally-Occurring Xenobiotics) to Predict Photoreaction Kinetics in Surface Freshwaters
Source: Molecules. 2019 Dec 18;25(1):9. doi: 10.3390/molecules25010009 (PMC7017383; doi:10.3390/molecules25010009)
Supplement: Supplementary file 1 [file molecules-25-00009-s001.zip › 3.2.4_gcc-4.4.0.zip!/3.2.4_gcc-4.4.0/3.2.4_gcc-4.4.0/doc/octave/html/interpreter/Creating-Sparse-Matrices-in-Oct_002dFiles.html]

Creating Sparse Matrices in Oct-Files - Untitled


Next: Using Sparse Matrices in Oct-Files,
Previous: Array and Sparse Differences,
Up: Sparse Matrices in Oct-Files


---

##### A.1.6.2 Creating Sparse Matrices in Oct-Files

You have several alternatives for creating a sparse matrix.
You can first create the data as three vectors representing the
row and column indexes and the data, and from those create the matrix.
Or alternatively, you can create a sparse matrix with the appropriate
amount of space and then fill in the values. Both techniques have their
advantages and disadvantages.

Here is an example of how to create a small sparse matrix with the first
technique

```
     int nz = 4, nr = 3, nc = 4;
     
     ColumnVector ridx (nz);
     ColumnVector cidx (nz);
     ColumnVector data (nz);
     
     ridx(0) = 0; ridx(1) = 0; ridx(2) = 1; ridx(3) = 2;
     cidx(0) = 0; cidx(1) = 1; cidx(2) = 3; cidx(3) = 3;
     data(0) = 1; data(1) = 2; data(2) = 3; data(3) = 4;
     
     SparseMatrix sm (data, ridx, cidx, nr, nc);
```

which creates the matrix given in section
Storage of Sparse Matrices. Note that the compressed matrix
format is not used at the time of the creation of the matrix itself,
however it is used internally.

As previously mentioned, the values of the sparse matrix are stored
in increasing column-major ordering. Although the data passed by the
user does not need to respect this requirement, the pre-sorting the
data significantly speeds up the creation of the sparse matrix.

The disadvantage of this technique of creating a sparse matrix is
that there is a brief time where two copies of the data exists. Therefore
for extremely memory constrained problems this might not be the right
technique to create the sparse matrix.

The alternative is to first create the sparse matrix with the desired
number of non-zero elements and then later fill those elements in. The
easiest way to do this is

```
     int nz = 4, nr = 3, nc = 4;
     SparseMatrix sm (nr, nc, nz);
     sm(0,0) = 1; sm(0,1) = 2; sm(1,3) = 3; sm(2,3) = 4;
```

That creates the same matrix as previously. Again, although it is not
strictly necessary, it is significantly faster if the sparse matrix is
created in this manner that the elements are added in column-major
ordering. The reason for this is that if the elements are inserted
at the end of the current list of known elements then no element
in the matrix needs to be moved to allow the new element to be
inserted. Only the column indexes need to be updated.

There are a few further points to note about this technique of creating
a sparse matrix. Firstly, it is possible to create a sparse matrix
with fewer elements than are actually inserted in the matrix. Therefore

```
     int nz = 4, nr = 3, nc = 4;
     SparseMatrix sm (nr, nc, 0);
     sm(0,0) = 1; sm(0,1) = 2; sm(1,3) = 3; sm(2,3) = 4;
```

is perfectly valid. However it is a very bad idea. The reason is that
as each new element is added to the sparse matrix the space allocated
to it is increased by reallocating the memory. This is an expensive
operation, that will significantly slow this means of creating a sparse
matrix. Furthermore, it is possible to create a sparse matrix with
too much storage, so having nz above equaling 6 is also valid.
The disadvantage is that the matrix occupies more memory than strictly
needed.

It is not always easy to know the number of non-zero elements prior
to filling a matrix. For this reason the additional storage for the
sparse matrix can be removed after its creation with the
maybe\_compress function. Furthermore, the maybe\_compress can
deallocate the unused storage, but it can equally remove zero elements
from the matrix. The removal of zero elements from the matrix is
controlled by setting the argument of the maybe\_compress function
to be true. However, the cost of removing the zeros is high because it
implies resorting the elements. Therefore, if possible it is better
is the user doesn't add the zeros in the first place. An example of
the use of maybe\_compress is

```
       int nz = 6, nr = 3, nc = 4;
     
       SparseMatrix sm1 (nr, nc, nz);
       sm1(0,0) = 1; sm1(0,1) = 2; sm1(1,3) = 3; sm1(2,3) = 4;
       sm1.maybe_compress ();  // No zero elements were added
     
       SparseMatrix sm2 (nr, nc, nz);
       sm2(0,0) = 1; sm2(0,1) = 2; sm(0,2) = 0; sm(1,2) = 0;
       sm1(1,3) = 3; sm1(2,3) = 4;
       sm2.maybe_compress (true);  // Zero elements were added
```

The use of the maybe\_compress function should be avoided if
possible, as it will slow the creation of the matrices.

A third means of creating a sparse matrix is to work directly with
the data in compressed row format. An example of this technique might
be


```
     octave_value arg;
     ...
     int nz = 6, nr = 3, nc = 4;   // Assume we know the max no nz
     SparseMatrix sm (nr, nc, nz);
     Matrix m = arg.matrix_value ();
     
     int ii = 0;
     sm.cidx (0) = 0;
     for (int j = 1; j < nc; j++)
       {
         for (int i = 0; i < nr; i++)
           {
             double tmp = foo (m(i,j));
             if (tmp != 0.)
               {
                 sm.data(ii) = tmp;
                 sm.ridx(ii) = i;
                 ii++;
               }
           }
         sm.cidx(j+1) = ii;
      }
     sm.maybe_compress ();  // If don't know a-priori
                            // the final no of nz.
```

which is probably the most efficient means of creating the sparse matrix.

Finally, it might sometimes arise that the amount of storage initially
created is insufficient to completely store the sparse matrix. Therefore,
the method `change_capacity` exists to reallocate the sparse memory.
The above example would then be modified as

```
     octave_value arg;
     ...
     int nz = 6, nr = 3, nc = 4;   // Assume we know the max no nz
     SparseMatrix sm (nr, nc, nz);
     Matrix m = arg.matrix_value ();
     
     int ii = 0;
     sm.cidx (0) = 0;
     for (int j = 1; j < nc; j++)
       {
         for (int i = 0; i < nr; i++)
           {
             double tmp = foo (m(i,j));
             if (tmp != 0.)
               {
                 if (ii == nz)
                   {
                     nz += 2;   // Add 2 more elements
                     sm.change_capacity (nz);
                   }
                 sm.data(ii) = tmp;
                 sm.ridx(ii) = i;
                 ii++;
               }
           }
         sm.cidx(j+1) = ii;
      }
     sm.maybe_mutate ();  // If don't know a-priori
                          // the final no of nz.
```

Note that both increasing and decreasing the number of non-zero elements in
a sparse matrix is expensive, as it involves memory reallocation. Also as
parts of the matrix, though not its entirety, exist as the old and new copy
at the same time, additional memory is needed. Therefore if possible this
should be avoided.
